# Anniversary of the New-York Academy of Medicine

**Published:** 1848-01

**Authors:** 


*Anniversary of the New-York Academy of Medicine.*—An anniversary address before the Academy was lately delivered, at the Broadway Tabernacle, by Dr. John W. Francis, before a very large auditory. Many persons, indeed, were prevented from gainning admittance, the hall being crowded to overflowing. This speaks well for a growing public interest in medical affairs, as well as for the personal reputation of the orator. The address was equal to the high expectations which had been entertained. It is to be published, so that members of the profession of a distance may have a share of the gratification afforded to those who enjoyed the privilege of being auditors on the interesting occasion.

